# Exploratory Analysis of Circulating GLP-1, GIP, and TMAO in Relation to Coronary Artery Disease Severity in Patients with Exertional Angina

**DOI:** 10.3390/biomedicines14020260

**Published:** 2026-01-23

**Authors:** Saime Batirel, Bengu Cetinkaya, Ali Sahin, Nodira Alakbarova, Tuba Guctekin, Beste Ozben, Mustafa Kürşat Tigen

**Affiliations:** 1 Department of Medical Biochemistry, School of Medicine, Marmara University, Istanbul 34854, Türkiye; 2 Genetic and Metabolic Diseases Research Center (GEMHAM), Marmara University, Istanbul 34854, Türkiye; 3 Department of Biochemistry (MED), Institute of Health Science, Marmara University, Istanbul 34854, Türkiyealakbarovan@bk.ru (N.A.); 4 Department of Cardiology, School of Medicine, Marmara University, Istanbul 34854, Türkiye

**Keywords:** coronary artery disease, glucagon-like peptide-1, glucose-dependent insulinotropic peptide, trimethylamine N-oxide, lipids

## Abstract

**Background/Objectives:** The gut–heart axis has garnered increasing attention. Incretin hormones such as glucagon-like peptide-1 (GLP-1) and glucose-dependent insulinotropic polypeptide (GIP), along with trimethylamine N-oxide (TMAO), have been implicated in the pathogenesis of coronary artery disease (CAD). This study aimed to investigate associations between plasma levels of GLP-1, GIP, and TMAO and the severity of CAD, alongside their correlations with serum biochemical parameters and fatty acid composition. **Methods:** Sixty-one patients undergoing coronary angiography were evaluated and stratified by Gensini scores into normal-coronary-artery, moderate-CAD, or severe-CAD groups. Biochemical parameters in serum and plasma GLP-1, GIP, and TMAO levels were measured. Plasma fatty acid composition was analyzed. **Results:** Fasting plasma GLP-1 and TMAO levels were not associated with CAD severity. Although GIP showed associations with CAD severity, these were not retained after adjustment for age and sex. Plasma myristic acid levels were positively associated with Gensini score. GLP-1 correlated positively with saturated fatty acids and negatively with monounsaturated fatty acids. TMAO levels inversely correlated with n-3 polyunsaturated fatty acids (PUFAs), particularly docosahexaenoic acid, and positively with the n-6/n-3 PUFA ratio, supporting its potential role in pro-atherogenic lipid profiles. **Conclusions:** These findings suggest complex associations between gut-derived metabolites, lipid metabolism, and CAD severity.

## 1. Introduction

Cardiovascular disease (CVD), particularly coronary artery disease (CAD), remains the leading cause of global morbidity and mortality. Beyond classical risk factors such as diabetes, dyslipidemia, hypertension, and chronic inflammation, recent research has highlighted the gut–heart axis as a critical component in CAD pathogenesis. Gut-derived molecules like glucagon-like peptide-1 (GLP-1), glucose-dependent insulinotropic peptide (GIP), and trimethylamine N-oxide (TMAO) have emerged as potential modulators of atherosclerosis through their effects on metabolism, endothelial function, and inflammation [1,2].

GLP-1 and GIP are incretin hormones secreted from intestinal L-cells and K-cells, respectively, and both enhance insulin secretion in a glucose-dependent manner. In addition, they have been shown to influence lipid metabolism, endothelial function, and inflammatory pathways, thereby potentially modulating atherosclerotic progression [2]. GLP-1, in particular, exerts vasoprotective effects by improving endothelial function and reducing inflammation [3,4]. It also contributes to improved lipid metabolism by reducing non-esterified fatty acids and lowering postprandial triglyceride levels [2]. These benefits have led to the widespread use of GLP-1 receptor agonists in type 2 diabetes treatment, especially because of their proven ability to reduce cardiovascular events in high-risk patients [5]. GIP may also influence cardiovascular function through several mechanisms. Studies have suggested that GIP modulates vasodilation via nitric oxide secretion and regulates vascular inflammation and leukocyte adhesion. Furthermore, GIP may impact lipid metabolism by promoting lipid uptake and processing in adipose tissue. However, in dysmetabolic states such as obesity and type 2 diabetes, GIP’s physiological effects may shift in a more atherogenic direction, raising questions about its dual role in cardiovascular health. While the therapeutic potential of GIP receptor agonism or antagonism in CVD remains under investigation, emerging evidence suggests that GIP may play a more complex and context-dependent role in atherosclerosis development [6].

Another key player in the gut–heart axis is TMAO. It is a microbial metabolite generated by the hepatic oxidation of trimethylamine (TMA), which is derived from the microbial metabolism of dietary choline, l-carnitine, and phosphatidylcholine [7]. TMAO has been implicated in various atherogenic mechanisms, including the promotion of inflammation, disruption of cholesterol metabolism, and enhancement of thrombogenic potential [8]. While TMAO has been associated with adverse cardiovascular outcomes, its causal role remains uncertain, particularly given evidence showing no significant contribution to early atherosclerosis in healthy populations. Further studies are warranted to clarify the role of microbiota-derived TMAO in atherogenesis [8]. Given its association with lipid metabolism and inflammation, TMAO may serve as a link between diet, gut microbiota, and cardiovascular risk [9,10].

While the individual effects of GLP-1, GIP, and TMAO on cardiovascular and metabolic health have been studied, their interrelationships and their associations with biochemical markers and plasma lipid composition remain insufficiently explored. Therefore, this exploratory cross-sectional study aimed to assess whether fasting plasma levels of GLP-1, GIP, and TMAO are associated with CAD severity, as determined by the Gensini score, in patients undergoing diagnostic coronary angiography. A secondary objective was to examine their correlations with routine biochemical parameters and plasma fatty acid composition to generate hypothesis-forming clinical insights regarding their potential relevance in the context of CAD.

## 2. Materials and Methods

### 2.1. Study Design and Participants

In this cross-sectional study, patients attending a cardiology department were recruited. Ethical approval was obtained from the Marmara University School of Medicine Ethics Committee, and all procedures were applied according to approved protocol (approval number: 09.2020.383, approval date: 3 April 2020). The privacy rights of the subjects were observed, and written informed consent was obtained from all participants.

Sixty-one consecutive patients were enrolled among those referred for clinically indicated coronary angiography because of exertional chest pain suggestive of myocardial ischemia. The indication for angiography was based on routine clinical decision-making, including symptom characteristics, cardiovascular risk profile, and non-invasive testing when necessary. Exclusion criteria were patient with severe comorbid conditions, such as end-stage renal disease or active malignancy, pregnant women, and patients under the age of 18.

At the time of angiography, a structured questionnaire was administered to the participants to obtain information on their lifestyle habits, medical history, and anthropometric measurements. Body mass index (BMI) was computed using the formula BMI (kg/m^2^) = Body weight (kg)/Height^2^ (m^2^).

### 2.2. Coronary Angiographic Data and Gensini Score Computation

Coronary angiography procedures were performed via trans-femoral or trans-radial access through 6 or 7 French diagnostic catheters according to current standard technique using a commercially available angiographic system (Artis Zee biplane; Siemens Healthcare, Erlangen, Germany). The extent and severity of coronary atherosclerosis among participants were quantitatively calculated using the Gensini score, which accounts for both the degree of lumen narrowing and the anatomical location of the narrowing [11]. Based on their Gensini scores, patients were categorized into three groups: Gensini 0 (normal coronary artery), Gensini 1–23 (moderate CAD), and Gensini > 23 (severe CAD). This categorization was based on both previously published thresholds and inspection of score distribution in the present cohort, which demonstrated a natural separation around a Gensini score of 23 [12]. Normal coronary arteries were defined by the absence of angiographically visible stenosis; however, undetected microvascular dysfunction cannot be excluded due to the lack of dedicated functional assessment.

### 2.3. Biological Sample Collection and Analyses of Routine Biochemical Parameters

Before the coronary angiography procedure, blood samples were collected from all patients after an overnight fasting period into tubes with and without EDTA-aprotinin. Fasting samples were chosen to ensure standardized metabolic conditions and to reduce variability related to recent food intake and gastric motility. This allowed for a standardized assessment of basal levels of the measured parameters. After plasma and serum were obtained, they were stored at –80 °C until analyses were performed. Serum glucose, insulin, triglyceride, high-density lipoprotein (HDL), and total cholesterol levels were determined using an automatic biochemistry analyzer (Beckman Coulter AU5800, Beckman Coulter, Inc., Brea, CA, USA). Subsequently, very low-density lipoprotein (VLDL) and Low-density lipoprotein (LDL) concentrations were calculated using the formul as (VLDL = Triglyceride/5), (LDL = Total cholesterol − HDL – VLDL). HOMA-IR was calculated as the ratio of fasting insulin to fasting glucose.

### 2.4. Measurements of GLP-1, GIP, and TMAO Levels in Plasma

Plasma levels of GLP-1, GIP, and TMAO were quantified using enzyme-linked immunosorbent assay (ELISA) kits (Elabscience, Wuhan, China; Sunred, Shanghai, China). The assays were performed according to the manufacturer’s instructions, and absorbance was measured using a microplate reader at 450 nm.

### 2.5. Fatty Acid Extraction and Analysis with GC–MS

Fatty acids were extracted from plasma samples according to Bligh and Dyer method [13]. These extracted fatty acids were then converted into fatty acid methyl esters (FAMEs) as described by Christie et al. The FAMEs were separated and analyzed using gas chromatography–mass spectrometry (GC–MS QP2010; Shimadzu Scientific Instruments, Kyoto, Japan) [14]. The FAMEWAX column was used during the analysis (30 m × 0.32 mm i.d., 0.25 µm film thickness; Restek, Bellefonte, PA, USA). The ion source temperature was set to 240 °C and the detector area temperature to 250 °C. Helium was used as the carrier gas at a flow rate of 3 mL/min. The injector temperature was set to 250 °C. The oven temperature was started from 130 °C and increased to 240 °C with an increase rate of 3 °C per minute and kept for 5 min. Samples were injected into the instrument at a 1:10 dilution and 1 µL volume. Fatty acid peaks formed as a result of the analysis were identified by matching them with FAME standards (The Food Industry 37 FAME mix, 35077 Restek Bellefonte, PA, USA). The amount of each fatty acid was expressed as a percentage of total plasma fatty acids. The percentages of total saturated fatty acids (SFAs), monounsaturated fatty acids (MUFAs), polyunsaturated fatty acids (PUFAs), n-6 PUFAs, n-3 PUFAs, and the n-6/n-3 PUFA ratio were calculated.

### 2.6. Statistical Analysis

Descriptive and inferential statistical analyses were performed using Jamovi software (version 2.3.26; Sydney, Australia). Ordinal logistic regression analyses were conducted using R software (version 4.4.1; R Foundation for Statistical Computing, Vienna, Austria), employing the ordinal, Brant, and VGAM packages.

Categorical variables were expressed as frequency (n) and percentage (%) and compared using the chi-square test. Continuous variables were presented as mean ± standard deviation (SD) for normally distributed data and as median with interquartile range (IQR, 25th–75th percentile) for non-normally distributed data. Normality and variance homogeneity were assessed using the Shapiro–Wilk and Levene’s tests, respectively. For two-group comparisons, the independent samples *t*-test or Mann–Whitney U test was used as appropriate. For comparisons involving three or more groups, one-way ANOVA (Fisher’s or Welch’s, depending on variance equality) or Kruskal–Wallis test was applied. Tukey’s HSD post hoc test was used for pairwise comparisons. Given the exploratory design and limited sample size, no additional multiple comparison corrections were performed in order to avoid excessive Type II error risk. Variables that were significant in univariate analyses were then included in an ordinal logistic regression model. Correlations were analyzed using Pearson’s or Spearman’s coefficients according to data distribution. A *p*-value < 0.05 was considered statistically significant.

## 3. Results

### 3.1. Baseline Characteristics of the Study Population

The study included 61 consecutive patients, of whom 33 were male and 28 were female (*p* = 0.786). The mean age was similar between the males and females, 59 ± 10 and 60 ± 13, respectively (*p* = 0.617). While BMI tended to be higher in females (31.95 ± 7.37) compared to males (29.75 ± 4.90), the difference was not statistically significant. It was found that males had significantly higher Gensini scores than females, indicating greater angiographic CAD severity in the male subgroup (median (IQR): 19.5 (6.6–45.0) vs. 6.0 (1.0–18.4), *p* = 0.013).

The baseline laboratory measurements showed median glucose of 114 (IQR: 103–159) mg/dL, insulin 11.39 (IQR: 7.00–17.50) mU/L, Homeostasis Model Assessment of Insulin Resistance (HOMA-IR) 3.37 (IQR: 1.97–5.83), triglycerides 127 (IQR: 87–196) mg/dL, VLDL 25 (IQR: 17–39), and LDL 108 (IQR: 90–145) mg/dL mg/dL, as well as mean HDL and total cholesterol levels of 40 ± 11 mg/dL and 187 ± 50 mg/dL, respectively. The cohort had median fasting GLP-1, GIP, and TMAO levels of 1.12 (IQR: 0.80–1.54) pg/mL, 208 (IQR: 129–379) pg/mL, and 2.55 (IQR: 2.23–3.02) ng/mL, respectively. Among these parameters, only GLP-1 levels showed a significant difference between the genders, being higher in females compared to males (*p* = 0.033).

Fatty acids with quantifiable serum concentrations and consistent detectability across samples were evaluated. For interpretability, the results are examined both individually and by metabolic class: SFAs, MUFAs, and PUFAs, including n-3 and n-6 subgroups. The n-6/n-3 ratio was also calculated due to its established cardiometabolic relevance. The most abundant plasma fatty acids detected in the plasma of all the patients were palmitic acid (43.42%), stearic acid (18.21%), linoleic acid (15.12%), oleic acid (8.62%), arachidonic acid (4.92%), docosahexaenoic acid (DHA) (1.90%), dihomo-γ-linolenic acid (DGLA) (1.40%), and myristic acid (1.26%). When aggregated by lipid class, SFAs constituted 65.25 ± 2.94%, MUFAs 10.71 ± 1.81%, and PUFAs 24.04 ± 2.27% of the total plasma fatty acids. The median n-6/n-3 PUFA ratio was 10.33 (IQR: 8.49–13.01).

### 3.2. Comparison of Patients According to Their Gensini Score

The patients participating in the study were statistically evaluated by dividing them into three groups according to their Gensini scores: those with normal coronary arteries (Gensini 0), those with moderate CAD (Gensini 1–23), and those with severe CAD (Gensini > 23) (Table 1). Of the patients included in the study, nine were in the normal-coronary-artery group, thirty-one were in the moderate-CAD group, and twenty-one were in the severe-CAD group. The proportion of male patients increased progressively with higher Gensini score categories. The patients in the moderate- and severe-CAD groups had higher smoking and alcohol consumption rates compared with the patients in the normal group. The frequencies of hypertension and diabetes were also higher in the higher-Gensini-score groups.

First, the associations between study variables and Gensini score categories were examined using univariate analyses. Variables showing significant differences across groups were subsequently selected for multivariable analyses.

In the univariate analyses (Table 1), the mean age was higher in both the moderate (*p* = 0.204) and severe (*p* = 0.130) CAD groups compared with the normal-coronary-artery group; however, these differences did not reach statistical significance. When the BMI values of the patients were examined according to these groups, the BMI values were numerically higher in the moderate-CAD group compared to the normal group (*p* = 0.194) and lower in the severe group compared to the moderate group (*p* = 0.057), although these differences did not reach statistical significance. It was observed that the GLP-1 and TMAO levels did not significantly differ across Gensini score categories, while the GIP levels differed significantly between groups, with the highest median values observed in the moderate-CAD group. Post hoc comparisons showed that GIP was significantly lower in severe vs. moderate CAD (*p* = 0.006), whereas the differences between normal vs. moderate (*p* = 0.181) and normal vs. severe (*p* = 0.596) were not statistically significant. In terms of fasting glucose, insulin, or HOMA-IR, no statistically significant differences were observed among the groups, although the insulin and HOMA-IR levels were highest in the moderate-CAD group. In terms of triglyceride, total cholesterol, VLDL, LDL, and HDL levels, there was no statistically significant difference between the three groups. The HDL levels tended to be lower and the VLDL levels tended to be higher in the higher-Gensini-score groups. The plasma fatty acid levels of the groups according to the Gensini scores are also presented in Table 1. When the groups were compared, there was no statistically significant difference between the groups in terms of total SFA, MUFA, and PUFA values. But a decreasing trend in total PUFAs was observed in the severe-CAD group compared to the other groups. This decrease was more pronounced in total n-6 PUFAs. When comparing individual fatty acids between the groups, differences were observed in the lauric acid and myristic acid levels. Notably, the levels of these fatty acids increased in parallel with higher Gensini scores. For myristic acid, post hoc analyses indicated a significant difference between the normal-coronary-artery group and the severe-CAD group (*p* = 0.016), whereas comparisons between the normal and moderate groups (*p* = 0.371) and between the moderate and severe groups (*p* = 0.074) did not reach statistical significance.

Those variables that were significant in the univariate analyses (BMI, GIP, and myristic acid) were then included in an ordinal logistic regression model adjusted for age and gender (Table 2). The proportional odds (PO) assumption was assessed using the Brant test, which indicated a violation of this assumption for the GIP variable (*p* = 0.006). Therefore, the analyses were performed using a partial proportional odds model (PPOM). The PPOM showed a significantly better fit than the conventional PO model, as confirmed by the likelihood ratio test (LR χ^2^ = 7.51, df = 1, *p* = 0.006). In the age- and gender-adjusted ordinal logistic regression analysis, myristic acid levels were independently and significantly associated with higher Gensini score categories (β = 1.254, *p* = 0.007). A one–standard deviation increase in myristic acid was associated with an approximately 3.5-fold increase in the odds of being in a higher Gensini score category (OR = 3.50, 95% CI: 1.25–9.81). Age demonstrated a borderline association with increasing Gensini score categories (OR = 1.07, 95% CI: 1.00–1.16, *p* = 0.061), indicating a trend toward higher CAD severity with advancing age, which is consistent with prior epidemiological evidence, whereas gender was not independently associated with Gensini score categories. In addition, plasma GIP levels were not independently associated with Gensini score categories after adjustment for age and gender and were excluded from the final model due to a violation of the proportional odds assumption and a lack of statistical significance.

### 3.3. Correlations of the Variables with the Gensini Scores of the Patients

Correlation analyses were conducted to evaluate the associations between Gensini scores and metabolic and biochemical parameters or fatty acid composition. Among the variables assessed, GIP demonstrated a significant inverse correlation with Gensini score, indicating that lower GIP levels tended to be observed with increasing angiographic disease severity. However, as shown in the age- and gender-adjusted ordinal logistic regression analysis presented above, this association did not persist as an independent predictor of Gensini score categories. HDL cholesterol also showed a significant inverse correlation with the Gensini score, consistent with its known protective role in atherosclerosis. In addition, a positive correlation was observed between the Gensini score and both lauric and myristic acid levels, supporting the findings from the Gensini-based subgroup analyses (Table 3).

### 3.4. Correlation Analysis of GLP-1, GIP, and TMAO

A Spearman correlation analysis was further performed to investigate the relationships between GLP-1, GIP, and TMAO levels and biochemical or fatty acid parameters. As summarized in Table 4, GLP-1 levels were positively associated with total SFAs and specifically with stearic and arachidic acids, while they were negatively correlated with total MUFAs and oleic acid. These results suggest a potential association between plasma fatty acid composition and GLP-1 levels, which may reflect complex bidirectional interactions between lipid metabolism and gut signaling. On the other hand, GIP did not show significant correlations with any metabolic or biochemical parameters, or with individual plasma fatty acids, except for a positive correlation trend with GLP-1. When examining the correlations with TMAO, the TMAO levels were found to be inversely correlated with the total n-3 PUFAs and DHA levels, and consequently positively correlated with the n-6/n-3 PUFA ratio, aligning with the hypothesis that TMAO may reflect pro-atherogenic metabolic states. Additionally, although not reaching statistical significance, the positive correlations observed between TMAO and triglycerides, total cholesterol, VLDL, and LDL levels were also noteworthy.

## 4. Discussion

In this study, we examined the relationship between circulating GLP-1, GIP, and TMAO levels and fatty acid composition and the serum biochemical parameters of patients with CAD. In the multivariable analyses, although BMI and GIP were associated with CAD severity in univariate analyses, only myristic acid retained an independent association with higher Gensini score categories after adjustment for age and sex. The correlation analyses further indicated that GLP-1 was positively associated with SFAs and negatively associated with MUFAs, while TMAO showed a negative correlation with n-3 PUFAs.

The development of CAD is influenced by both non-modifiable factors such as age, gender, and genetic predisposition and modifiable risk factors, including smoking, physical inactivity, unhealthy dietary habits, hypertension, type 2 diabetes, dyslipidemia, and obesity [15]. Consistent with the previous literature, in this study, male patients exhibited significantly higher Gensini scores. On the other hand, the relationship between obesity and CVD outcomes remains controversial due to the so-called obesity paradox, which suggests that excess weight may confer a survival advantage in individuals with established CVD. Several mechanisms have been proposed to explain this, including the protective effects of adipose tissue-derived hormones and cytokines and the greater metabolic reserves in obese individuals that may enhance resilience during catabolic stress [16,17]. In this study, the BMI values appeared to be higher in patients with moderate CAD compared with those without angiographically evident CAD, while lower BMI values were observed in the severe-CAD group. However, this pattern did not persist as an independent association after multivariable adjustment and should therefore be interpreted with caution. Meanwhile, plasma myristic acid, a minor SFA, emerged as the only fatty-acid-related parameter independently associated with higher angiographic CAD burden in our age- and sex-adjusted ordinal model, suggesting that this specific SFA may capture clinically relevant variations in atherosclerotic severity. This observation is biologically plausible as myristic acid has been highlighted as a particularly cholesterol-raising SFA in prior work [18], and large prospective cohorts have reported a positive association between dietary myristic acid intake and incident coronary heart disease [19]. In addition, it has been reported to be elevated in patients with coronary atherosclerosis compared with controls [20]. Taken together, our findings extend the existing literature by linking circulating myristic acid to angiographic CAD severity quantified by the Gensini score. Moreover, in our study, the triglyceride and VLDL levels were numerically higher in the severe-CAD group, while the HDL cholesterol levels tended to be lower with increasing CAD severity. In addition, the total and n-6 PUFA levels were lower in patients with higher Gensini scores. Taken together, these findings suggest a descriptive pattern of a less favorable lipid profile accompanying more advanced coronary atherosclerosis, in line with previous reports [21,22]. However, as these trends did not reach statistical significance, they should be interpreted cautiously.

Recent studies highlight the role of gut microbiota-derived molecules, particularly GLP-1, GIP, and TMAO, in CVD development, supporting the gut–heart axis concept [23]. GLP-1 and GIP are secreted in response to oral glucose intake, stimulate insulin secretion through the incretin effect, and also exert extra-pancreatic actions that may help to prevent cardiovascular complications, as shown in recent studies [24,25].

GLP-1 has attracted increasing interest for its cardiovascular effects beyond glucose regulation. Through GLP-1 receptor activation in vascular and cardiac tissues, it can improve endothelial function, enhance ischemia tolerance, suppress inflammation, and stabilize atherosclerotic plaques, particularly at pharmacological levels [24,26]. Yuan et al. [27] and Nagase et al. [28] reported inverse associations between serum GLP-1 levels and Gensini scores, suggesting that reduced GLP-1 may indicate more severe CAD. However, in our study, GLP-1 levels were not significantly associated with CAD severity. Furthermore, no significant correlations were observed with glucose, insulin, or lipid parameters. Interestingly, GLP-1 levels correlated positively with SFAs and negatively with MUFAs. This aligns with experimental evidence showing that stearic acid, a representative SFA, stimulates GLP-1 secretion via GPR120-mediated PLCβ–PKC–IP3R signaling [29]. However, our result contrasts with in vitro findings where oleic acid, a major MUFA, stimulates GLP-1 secretion by enhancing cellular metabolism, suggesting potential differences between in vitro and in vivo regulation [30]. These differences may reflect varying regulatory mechanisms in vitro and in human physiology.

Although GIP is known for its insulinotropic effects, recent studies highlight its involvement in lipid metabolism, inflammation, and cardiovascular regulation, although the findings remain inconsistent [2]. Preclinical data suggest anti-atherosclerotic roles via reduced foam cell formation, macrophage infiltration, and improved plaque stability [31,32], supported by GIP receptor expression in adipose, endothelial, and cardiac tissues [25]. Conversely, some studies report pro-atherogenic effects, including lipid accumulation and upregulation of osteopontin and endothelin-1 [6]. In our study, aside from the univariate findings, plasma GIP levels were not significantly correlated with fasting glucose, insulin, HOMA-IR, lipid parameters, or individual fatty acids. This may be related to the predominantly post-prandial physiology of GIP, which is not fully captured under fasting conditions. Experimental studies in murine models suggest potential anti-atherosclerotic effects of GIP that are lost upon inhibition of GIP receptor signaling, highlighting the complexity of its vascular actions [32]. Elevated GIP in peripheral artery disease patients has also been noted, although not consistently in CAD [31]. On the other hand, GIP’s lack of correlation with fasting lipids may be due to its predominant role in postprandial lipid handling, especially under insulin-sufficient conditions [33].

Although both GLP-1 and GIP are incretin hormones, the coordination of their secretion and systemic effects is not yet fully understood. In our study, GLP-1 and GIP levels showed a positive, although non-significant, correlation, consistent with previous findings that they are co-regulated but not tightly coupled. A weak but significant correlation between GLP-1 and GIP responses to oral glucose was reported in non-diabetic individuals [34], while, in another study, a stronger relationship was observed in healthy individuals and first-degree relatives of patients with type 2 diabetes [35]. This high interindividual variability suggests that their regulation may differ depending on metabolic status, gastrointestinal function, or receptor sensitivity [25]. Additionally, circulating GLP-1 and GIP levels are often concurrently low, suggesting a coordinated but indirect regulation rather than direct hormonal stimulation [25]. The receptors of both hormones are co-expressed in pancreatic β-cells and cardiovascular tissues, and dual agonists like tirzepatide target both pathways and have demonstrated superior glycemic and weight-lowering effects compared to GLP-1 monotherapy, highlighting the therapeutic potential of this synergistic approach. These observations have led to increasing interest in dual agonists such as tirzepatide; however, their role in CVD management requires further clinical confirmation [2].

TMAO, a gut microbiota-derived metabolite, has been implicated in cardiovascular risk, especially atherosclerosis. It is formed when gut microbes metabolize nutrients like choline and carnitine into TMA, which is then oxidized in the liver by flavin-containing monooxygenase 3 (FMO3) [36]. Animal studies showed that TMAO promotes atherosclerotic lesion formation, foam cell accumulation, and platelet reactivity. However, the human data remain inconsistent [8]. In our study, TMAO showed non-significant positive correlations with triglycerides, cholesterol, and VLDL but was inversely correlated with total n-3 PUFAs, particularly DHA, and positively with the n-6/n-3 ratio, indicating a link to pro-inflammatory lipid profiles. These observations are compatible with previous reports proposing that TMAO may contribute to inflammatory and dyslipidemic pathways [8,36]. It should be noted, however, that circulating TMAO levels are strongly influenced by habitual diet and gut microbiota composition [37], which were not directly assessed in this study and may have contributed to interindividual variability. Moreover, given the cross-sectional design, mechanistic inferences cannot be drawn. Animal studies also indicate that TMAO may impair reverse cholesterol transport, promote foam cell formation, and exacerbate endothelial dysfunction [9].

This study has several limitations: (1) The cross-sectional and exploratory design precludes causal inference; therefore, the observed associations should be interpreted as associative and hypothesis-generating rather than mechanistic or predictive. (2) In our study, patients were recruited from a symptomatic population referred for invasive coronary angiography due to a high pretest probability of CAD. Because invasive coronary angiography is typically reserved for patients with suspected or established disease, the number of individuals with normal coronary arteries was relatively small, which may have limited the study’s statistical power and generalizability. This imbalance may have attenuated, rather than exaggerated, the observed group differences. (3) Patient classification was based on Gensini scoring, which reflects epicardial coronary anatomy only. Coronary microvascular dysfunction was not routinely assessed during invasive coronary angiography in our catheterization laboratory. Therefore, patients classified as having “normal coronary arteries” may still have functional coronary abnormalities or underlying microvascular coronary disease. This potential misclassification should be considered when interpreting comparisons across Gensini-based groups. (4) Fasting blood samples were used to provide standardized metabolic conditions and assess basal incretin levels. However, as GLP-1 and GIP are predominantly post-prandial hormones, fasting measurements may not fully capture their dynamic physiological responses. Future studies incorporating standardized meal challenges or post-prandial testing may provide complementary insights. (5) TMAO concentrations are influenced by dietary intake and gut microbiota composition, which were not directly assessed in this study. (6) Lifestyle factors and background cardiovascular medications were recorded but not controlled for in the analyses. Consequently, residual confounding cannot be excluded. (7) Participants were recruited from a referral population undergoing clinically indicated coronary angiography, which may introduce selection bias.

## 5. Conclusions

This cross-sectional study highlights associations between gut-derived metabolites and plasma lipid composition in patients with CAD. Although GIP showed univariate associations with disease severity, these were not retained after multivariable adjustment. In contrast, the plasma myristic acid levels remained independently associated with higher Gensini score categories after age- and sex-adjusted analyses. GLP-1 showed correlations with SFAs and MUFAs, and TMAO was associated with a lipid profile characterized by lower n-3 PUFA levels. Collectively, these findings point to complex and context-dependent interactions between gut-derived metabolites, lipid metabolism, and coronary atherosclerosis and should be viewed as hypothesis-generating.

## Figures and Tables

**Table 1:** Evaluation of patients according to Gensini score.

Variable	Normal Coronary Artery (Gensini 0)	Moderate CAD (Gensini 1–23)	Severe CAD (Gensini > 23)	*p*
Number of patient (male/female) (n)	3/6	15/16	15/6	0.105
Lifestyle factors				
Smoking (*n*) (%)	2/9 (22%)	10/31 (32%)	7/21 (33%)	0.819
Alcohol consumption (*n*) (%)	1/9 (11%)	4/31 (13%)	4/21 (19%)	0.784
Clinical conditions				
Hypertension (*n*) (%)	6/9 (67%)	22/31 (71%)	18/21 (86%)	0.386
Diabetes (*n*) (%)	2/9 (22%)	16/31 (52%)	12/21 (57%)	0.200
Medications				
Anti-diabetic (*n*) (%)	1/9 (11%)	9/31 (29%)	9/21 (43%)	0.213
Anti-platelet (*n*) (%)	8/9 (89%)	29/31 (94%)	20/21 (95%)	0.812
Anti-hypertensive (*n*) (%)	4/9 (44%)	18/31 (58%)	14/21 (67%)	0.519
Statins (*n*) (%)	8/9 (89%)	28/31 (90%)	19/21 (90%)	0.990
Age (years)	53 ± 13	60 ± 11	62 ± 11	0.143
BMI (kg/m^2^)	28.52 ± 5.27	32.65 ± 6.45	28.58 ± 5.16	**0.040**
Gensini score	0 (0–0)	6.0 (3.5–10.5)	45.0 (37.5–63.0)	**<0.001**
GLP-1 (pg/mL)	1.12 (1.00–1.23)	1.11 (0.81–1.52)	1.16(0.55–1.60)	0.961
GIP (pg/mL)	259 (195–295)	374 (197–484)	143 (112–206)	**0.020**
TMAO (ng/mL)	2.47 (2.39–2.63)	2.70 (2.30–4.11)	2.43 (2.12–2.70)	0.246
Glucose (mg/dL)	110 (88–126)	114 (105–149)	117 (103–166)	0.496
Insulin (mU/L)	7.99 (4.58–13.20)	15.09 (7.52–22.20)	9.93 (7.59–13.30)	0.111
HOMA-IR	2.85 (1.04–3.45)	4.83 (2.44–7.31)	2.86 (1.95–3.92)	0.074
Triglyceride (mg/dL)	99 (87–110)	128 (87–165)	149 (94–204)	0.190
Total cholesterol (mg/dL)	184 ± 35	195 ± 58	178 ± 44	0.496
VLDL (mg/dL)	20 (17–22)	26 (17–33)	30 (19–41)	0.190
LDL (mg/dL)	115 (90–145)	115 (91.5–157)	107 (87–130)	0.596
HDL (mg/dL)	43 ± 10	42 ± 10	35 ± 11	0.075
SFAs (%)	65.27 (64.48–66.38)	64.46 (62.01–67.09)	64.90 (63.61–67.92)	0.357
Lauric acid (12:0)	0.15 (0.13–0.17)	0.15 (0.12–0.20)	0.21 (0.14–0.35)	0.113
Myristic acid (14:0)	1.05 ± 0.25	1.21 ± 0.30	1.42 ± 0.38	**0.012**
Palmitic acid (16:0)	43.68 ± 2.06	42.97 ± 2.71	43.98 ± 2.35	0.350
Margaric acid (17:0)	0.70 ± 0.14	0.63 ± 0.12	0.69 ± 0.14	0.129
Stearic acid (18:0)	18.21 ± 2.63	18.34 ± 1.54	18.02 ± 2.27	0.851
Arachidic acid (20:0)	0.23 (0.22–0.25)	0.21 (0.19–0.25)	0.22 (0.21–0.25)	0.343
Behenic acid (22:0)	0.47 ± 0.12	0.42 ± 0.12	0.41 ± 0.14	0.577
Lignoceric acid (24:0)	0.29 (0.25–0.33)	0.26 (0.22–0.36)	0.29 (0.26–0.37)	0.605
MUFAs	10.30 ± 1.64	10.62 ± 1.85	11.01 ± 1.85	0.576
Palmitoleic acid (16:1)	0.65 (0.48–0.86)	0.68 (0.45–0.83)	0.69 (0.44–0.91)	0.951
Oleic acid (18:1)	8.03 ± 1.27	8.59 ± 1.56	8.92 ± 1.63	0.359
PUFAs	24.31 ± 1.38	24.59 ± 2.41	23.12 ± 2.12	0.063
Linoleic acid (18:2)	14.83 ± 1.96	15.77 ± 2.64	14.26 ± 2.22	0.090
DGLA (20:3)	1.33 ± 0.35	1.42 ± 0.37	1.40 ± 0.41	0.841
Arachidonic acid (20:4)	5.44 ± 1.37	4.82 ± 1.17	4.84 ± 0.82	0.305
DHA (22:6)	2.09 (1.61–2.59)	1.74 (1.36–2.07)	1.82 (1.48–2.24)	0.686
Total n-6 PUFAs	21.96 ± 1.61	22.40 ± 2.49	20.90 ± 2.14	0.074
Total n-3 PUFAs	2.29 (1.76–2.83)	2.04 (1.74–2.31)	2.27 (1.67–2.60)	0.682
n-6/n-3 PUFAs	9.43 (7.46–13.01)	10.99 (9.66–14.00	9.81 (7.90–11.91)	0.324

CAD, coronary artery disease; BMI, body mass index; GLP-1, glucagon-like peptide-1; GIP, glucose-dependent insulinotropic peptide; TMAO, trimethylamine N-oxide; HOMA-IR, Homeostasis Model Assessment of Insulin Resistance; VLDL, very low-density lipoprotein; LDL, low-density lipoprotein; HDL, high-density lipoprotein; SFAs, saturated fatty acids; MUFAs, monounsaturated fatty acids; PUFAs, polyunsaturated fatty acids; DGLA, dihomo gamma linolenic acid; DHA, docosahexaenoic acid; SD, standard deviation; IQR, interquartile range. Data are presented as mean ± SD for normally distributed variables and as median (IQR) for non-normally distributed variables. Group comparisons were performed using one-way ANOVA (Fisher’s or Welch’s, as appropriate) for parametric variables and Kruskal–Wallis test for non-parametric variables. Bold *p*-values indicate statistical significance at *p* < 0.05.

**Table 2:** Age- and gender-adjusted ordinal logistic regression analysis of factors associated with higher Gensini score categories.

Variable	β	OR	%95 CI	*p*
Age	0.072	1.07	1.00–1.16	0.061
Gender	1.158	3.18	0.72–13.99	0.126
BMI	−0.058	0.94	0.84–1.07	0.351
GIP	-----	-----	-----	>0.05
Myristic acid	1.254	3.50	1.25–9.81	**0.007**

BMI, body mass index; GIP, glucose-dependent insulinotropic peptide; β, coefficient; OR, odds ratio; CI, confidence interval. Age- and gender-adjusted ordinal logistic regression analysis was performed using a partial proportional odds model due to violation of the proportional odds assumption (Brant test *p* = 0.006). Variables included were selected based on univariate significance. Results are presented as β coefficients, ORs, and 95% CIs. Bold *p*-values indicate statistical significance at *p* < 0.05.

**Table 3:** Correlations of the variables with Gensini scores.

Variable	Gensini Score	
	Spearman’s Rho	*p*-Value
Age (years)	0.165	0.203
BMI (kg/m^2^)	−0.200	0.137
GLP1 (pg/mL)	−0.036	0.783
GIP (pg/mL)	−0.332	**0.034**
TMAO (ng/mL)	−0.184	0.249
Glucose (mg/dL)	0.199	0.137
Insulin (mU/L)	0.030	0.829
HOMA-IR	0.086	0.527
Triglyceride (mg/dL)	0.217	0.105
Total cholesterol (mg/dL)	−0.107	0.427
VLDL (mg/dL)	0.217	0.105
LDL (mg/dL)	−0.157	0.243
HDL (mg/dL)	−0.330	**0.012**
SFAs (%)	0.077	0.553
Lauric acid (12:0)	0.205	0.112
Myristic acid (14:0)	0.311	**0.015**
Palmitic acid (16:0)	0.067	0.607
Margaric acid (17:0)	0.100	0.445
Stearic acid (18:0)	−0.013	0.922
Arachidic acid (20:0)	−0.035	0.790
Behenic acid (22:0)	−0.124	0.340
Lignoceric acid (24:0)	0.160	0.219
MUFAs (%)	0.060	0.646
Palmitoleic acid (16:1)	−0.061	0.642
Oleic acid (18:1)	0.136	0.297
PUFAs (%)	−0.159	0.221
Linoleic acid (18:2)	−0.136	0.298
DGLA (20:3)	0.021	0.871
Arachidonic acid (20:4)	−0.091	0.485
DHA (22:6)	0.036	0.784
Total n-6 PUFAs	−0.177	0.172
Total n-3 PUFAs	0.073	0.575
n-6/n-3 PUFAs	−0.126	0.333

BMI, body mass index; GLP-1, glucagon-like peptide-1; GIP, glucose-dependent insulinotropic peptide; TMAO, trimethylamine N-oxide; HOMA-IR, Homeostasis Model Assessment of Insulin Resistance; VLDL, very low-density lipoprotein; LDL, low-density lipoprotein; HDL, high-density lipoprotein; SFAs, saturated fatty acids; MUFAs, monounsaturated fatty acids; PUFAs, polyunsaturated fatty acids; DGLA, dihomo gamma linolenic acid; DHA, docosahexaenoic acid. Data are presented as correlation coefficients (Spearman’s ρ) and corresponding *p*-values. Spearman’s rank correlation test was used to assess associations between Gensini score and the variables. Bold *p*-values indicate statistical significance at *p* < 0.05.

**Table 4:** Correlations of the variables with GLP-1, GIP, and TMAO.

Variable	GLP1 (pg/mL) *n* = 61		GIP (pg/mL) *n* = 41		TMAO (ng/mL) *n* = 41	
	Spearman’s Rho	*p*-Value	Spearman’s Rho	*p*-Value	Spearman’s Rho	*p*-Value
Age (years)	0.136	0.295	−0.082	0.612	−0.245	0.123
BMI (kg/m^2^)	0.181	0.179	0.201	0.219	0.142	0.374
GLP1 (pg/mL)	-	-	0.277	0.079	−0.035	0.828
GIP (pg/mL)	0.277	0.079	-	-	0.185	0.319
TMAO (ng/mL)	−0.035	0.828	0.185	0.319	-	-
Glucose (mg/dL)	0.014	0.920	−0.171	0.286	−0.058	0.720
Insulin (mU/L)	0.146	0.294	−0.045	0.785	0.195	0.228
HOMA-IR	0.129	0.338	−0.054	0.737	0.111	0.486
Triglyceride (mg/dL)	−0.050	0.710	0.046	0.773	0.271	0.086
Total cholesterol (mg/dL)	−0.045	0.740	0.260	0.101	0.300	0.057
VLDL (mg/dL)	−0.050	0.710	0.046	0.773	0.271	0.086
LDL (mg/dL)	−0.059	0.663	0.278	0.078	0.250	0.115
HDL (mg/dL)	0.104	0.440	0.108	0.502	0.149	0.353
SFAs (%)	0.326	**0.010**	0.011	0.944	0.079	0.624
Lauric acid (12:0)	−0.076	0.562	−0.105	0.511	−0.026	0.870
Myristic acid (14:0)	0.033	0.803	−0.136	0.394	0.070	0.662
Palmitic acid (16:0)	0.161	0.216	−0.002	0.991	0.047	0.770
Margaric acid (17:0)	0.090	0.488	−0.118	0.461	−0.205	0.198
Stearic acid (18:0)	0.238	0.065	0.095	0.551	0.129	0.419
Arachidic acid (20:0)	0.302	**0.018**	−0.161	0.314	−0.080	0.619
Behenic acid (22:0)	0.102	0.433	−0.132	0.410	0.074	0.644
Lignoceric acid (24:0)	−0.024	0.853	−0.230	0.148	−0.028	0.862
MUFAs (%)	−0.264	**0.040**	−0.027	0.867	−0.187	0.240
Palmitoleic acid (16:1)	−0.011	0.932	0.144	0.366	−0.082	0.611
Oleic acid (18:1)	−0.270	**0.035**	−0.085	0.594	−0.194	0.224
PUFAs (%)	−0.190	0.143	0.031	0.847	0.024	0.883
Linoleic acid (18:2)	−0.074	0.570	0.060	0.708	0.147	0.357
DGLA (20:3)	−0.034	0.793	0.132	0.410	0.037	0.819
Arachidonic acid (20:4)	−0.113	0.386	−0.105	0.513	−0.104	0.516
DHA (22:6)	−0.066	0.614	−0.115	0.471	−0.384	**0.014**
Total n-6 PUFAs	−0.179	0.167	0.049	0.762	0.159	0.321
Total n-3 PUFAs	−0.111	0.394	−0.135	0.399	−0.391	**0.012**
n-6/n-3 PUFAs	0.049	0.710	0.187	0.242	0.372	**0.017**

BMI, body mass index; GLP-1, glucagon-like peptide-1; GIP, glucose-dependent insulinotropic peptide; TMAO, trimethylamine N-oxide; HOMA-IR, Homeostasis Model Assessment of Insulin Resistance; VLDL, very low-density lipoprotein; LDL, low-density lipoprotein; HDL, high-density lipoprotein; SFAs, saturated fatty acids; MUFAs, monounsaturated fatty acids; PUFAs, polyunsaturated fatty acids; DGLA, dihomo gamma linolenic acid; DHA, docosahexaenoic acid. Data are presented as correlation coefficients (Spearman’s ρ) and corresponding *p*-values. Spearman’s rank correlation test was used to assess associations between GLP-1, GIP, and TMAO levels and the variables. Bold *p*-values indicate statistical significance at *p* < 0.05.

## Data Availability

The datasets used and/or analyzed during the current study are available from the corresponding author upon reasonable request.

## Abbreviations

The following abbreviations are used in this manuscript:

BMI	Body mass index
CAD	Coronary artery disease
CI	Confidence interval
CVD	Cardiovascular disease
DGLA	Dihomo gamma linolenic acid
DHA	Docosahexaenoic acid
FAME	Fatty acid methyl ester
FMO	Flavin-containing monooxygenas
GIP	Glucose-dependent insulinotropic peptide
GLP-1	Glucagon-like peptide-1
HDL	High-density lipoprotein
HOMA-IR	Homeostasis Model Assessment of Insulin Resistance
IQR	Interquartile range
LDL	Low-density lipoprotein
MUFAs	Monounsaturated fatty acids
OR	Odds ratio
PO	Proportional odds
PPOM	Partial proportional odds model
PUFAs	Polyunsaturated fatty acids
SD	Standard deviation
SFAs	Saturated fatty acids
TMA	Trimethylamine
TMAO	Trimethylamine N-oxide
VLDL	Very low-density lipoprotein
